# To: Critical COVID-19 and neurological dysfunction - a direct comparative analysis between SARS-CoV-2 and other infectious pathogens

**DOI:** 10.5935/2965-2774.20230383-en

**Published:** 2023

**Authors:** Rohan Magoon, Varun Suresh

**Affiliations:** 1 Department of Anaesthesia, Atal Bihari Vajpayee Institute of Medical Sciences and Dr. Ram Manohar Lohia Hospital - Baba Kharak Singh Marg, New Delhi, India; 2 Department of Anesthesia and Intensive Care, Jaber Al Ahmad Al Sabah Hospital -Arabian Gulf, Kuwait

## To the Editor,

Teixeira-Vaz et al. deserve applause for staging a prospective analysis of neurological dysfunction ensuing after infection from severe acute respiratory syndrome coronavirus 2 (SARS-CoV-2) *versus* other pathogens.^(1)^ While the authors show that critically ill coronavirus disease 2019 (COVID-19) patients are prone to neurological complications, there is a need to consider additional factors in interpreting their research findings.

The index comparison revealed that the COVID-19 group was under sedoanalgesia for a significantly longer duration than the non-COVID-19 group (p = 0.025, with n = 27 in each group). Despite the days under sedoanalgesia not emerging as a factor associated with neurological complications in the small sample-sized univariate analysis conducted by Teixeira-Vaz et al., it remains difficult to draw any meaningful inferences that fall short of knowledge on the nature of sedation.^(1)^ The former becomes important when the systematic literature links benzodiazepines with an accentuated risk of *delirium* and dexmedetomidine with an attenuated risk of *delirium* in critically ill patients.^(2,3)^ Fraser et al. also suggested increased mechanical ventilation and length of intensive care unit (ICU) stay with benzodiazepine sedation.^(4)^ The described parameters, even in the study by Teixeira-Vaz et al., could likely have been affected by variables beyond the nature of the underlying disease (SARS-CoV-2 or other infections) unless some protocolized management approach, such as the ABCDEF bundle, was followed by the research group.^(1,2)^

Regarding more specific evidence, COVID-D (a multicenter cohort study by Pun et al. across 69 ICUs in 14 countries that included 2088 COVID-19 patients) outlined benzodiazepine use as a modifiable risk factor for *delirium*.^(2)^ Furthermore, the investigators retrospectively discussed the role of preexisting alcoholic status and the use of antipsychotics in their study, the lack of which is hard to overlook in the prospective research endeavor by Teixeira-Vaz et al.^(1,2)^

However, the remarkable 1.98-fold higher risk of composite neurological complications delineated in the COVID-19 cohort of the Teixeira-Vaz et al. study buttresses the crucial role of neuroinflammation in dictating the outcomes.^(1)^ Herein, it would be worthwhile to elucidate that Saxena et al. recently reported significantly higher fibrinogen levels in COVID-19 patients with an altered cognitive state during the ICU stay: 7.1g/L (6.58 - 8.30g/L) as opposed to 6.63g/L (5.41 - 7.77g/L) in those without any cognitive alteration (Wilcoxon p value = 0.026), suggesting its greater role among all other routine inflammatory markers in SARS-CoV-2 neuro-prognostication. ^(5)^
